# A target enrichment approach for enhanced recovery of *Synchytrium endobioticum* nuclear genome sequences

**DOI:** 10.1371/journal.pone.0296842

**Published:** 2024-02-12

**Authors:** Hai D. T. Nguyen, Ekaterina Ponomareva, Kasia Dadej, Donna Smith, Melissa Antoun, Theo A. J. van der Lee, Bart T. L. H. van de Vossenberg

**Affiliations:** 1 Ottawa Research and Development Centre, Agriculture and Agri-Food Canada, Ottawa, Ontario, Canada; 2 Charlottetown Laboratory, Canadian Food Inspection Agency, Charlottetown, Prince Edward Island, Canada; 3 Biointeractions and Plant Health, Wageningen University and Research, Wageningen, The Netherlands; 4 Netherlands Institute for Vectors, Invasive Plants and Plant Health, Dutch National Plant Protection Organization, Wageningen, the Netherlands; Chandigarh University, INDIA

## Abstract

Potato wart disease is caused by the obligate fungal pathogen *Synchytrium endobioticum*. DNA extraction from compost, purified spores and crude wart tissue derived from tuber galls of infected potatoes often results in low *S*. *endobioticum* DNA concentration or highly contaminated with DNA coming from other microorganisms and the potato host. Therefore, Illumina sequencing of these samples generally results in suboptimal recovery of the nuclear genome sequences of *S*. *endobioticum*. A hybridization-based target enrichment protocol was developed to strongly enhance the recovery of *S*. *endobioticum* DNA while off-target organisms DNA remains uncaptured. The design strategy involved creating a set of 180,000 molecular baits targeting both gene and non-gene regions of *S*. *endobioticum*. The baits were applied to whole genome amplified DNA samples of various *S*. *endobioticum* pathotypes (races) in compost, from purified spores and crude wart tissue samples. This was followed by Illumina sequencing and bioinformatic analyses. Compared to non-enriched samples, target enriched samples: 1) showed a significant increase in the proportion of sequenced bases mapped to the *S*. *endobioticum* nuclear genome, especially for crude wart tissue samples; 2) yielded sequencing data with higher and better nuclear genome coverage; 3) biased genome assembly towards *S*. *endobioticum* sequences, yielding smaller assembly sizes but higher representation of putative *S*. *endobioticum* contigs; 4) showed an increase in the number of *S*. *endobioticum* genes detected in the genome assemblies. Our hybridization-based target enrichment protocol offers a valuable tool for enhancing genome sequencing and NGS-based molecular detection of *S*. *endobioticum*, especially in difficult samples.

## Introduction

*Synchytrium endobioticum* is an obligate fungal plant pathogen that causes potato wart disease. This pathogen is responsible for the development of wart-like growths on the tubers, stems, and stolons of infected plants. Infected potato tubers lose their market value, as the warts make them unattractive for both consumers and processors. Also, the warted tissue tends to rot quite rapidly, making it unfit for human consumption. The pathogen’s ability to persist in soil and survive harsh environmental conditions makes it a threat to potato cultivation worldwide. Furthermore, it is also considered a quarantine pathogen in many countries in the world, and outbreaks can result in severe economic losses due to regulatory restrictions and market closures. There are more than 40 pathotypes (races) of *S*. *endobioticum*, each characterized by their ability to infect to a specific set of potato varieties. Pathotypes 1(D1), 2(G1), 6(O1), and 18(T1) are currently considered the most prevalent [1].

Target enrichment is a molecular biology protocol that involves the specific capture of DNA fragments corresponding to the regions of interest, followed by amplification and sequencing. Recently, target enrichment is being applied to study some obligate plant pathogens, such as the population genetics of *Pseudoperonospora* (downy mildews) [2], and the phylogeny of various plant pathogenic oomycetes from herbarium samples [3]. When some of us were working on the genome sequencing and characterization of *S*. *endobioticum* [4], even if the spores were isolated in a pure fraction, a large number of sequences from the potato host and other microorganisms would be obtained in the next generation sequencing (NGS) runs, causing a significant reduction in the yield of reads belonging to *S*. *endobioticum*. Sufficient sequencing coverage on the smaller mitochondrial genome of *S*. *endobioticum* was obtained in the previous study [5], but there was poor sequencing coverage of its nuclear genome. Sufficient coverage of the nuclear genome is essential for genome characterization, evaluation of nuclear encoded genes and their function, in particular effectors that are linked to pathotype identity [6].

In this study, we designed molecular baits using the reference genomes of *S*. *endobioticum* characterized previously [4]. We then tested a target enrichment protocol (more specifically a hybridization-based capture method) for enhancing the recovery of *S*. *endobioticum* nuclear genome sequences from Illumina library preparations. The aim of our study is to develop a method to enhance sequencing the nuclear genome of *S*. *endobioticum*, which can also be used to improve NGS-based molecular detection methods, where the starting amount of *S*. *endobioticum* DNA is low or from highly contaminated samples.

## Materials and methods

### Bait design

A customized bait set was designed in consultation with Daicel Arbor Biosciences. Briefly, genomes of two *S*. *endobioticum* isolates previously characterized [4] were downloaded: *S*. *endobioticum* MB42 (NCBI GenBank Accession No. QEAN00000000.1) and *S*. *endobioticum* LEV6574 (NCBI Accession No. QEAM00000000.1). Gene coordinates, that include both introns and exons, were extracted. Baits of 80 nt long at 0.75× tiling density (where an 80 nt bait starts at every ~120 bp) resulted in 127,286 baits for MB42 and 129,880 baits for LEV6547, combined for a total of 257,166 baits. These baits were checked for homology against the potato genome (*Solanum tuberosum* assembly SolTub_3.0 from European Nucleotide Archive Accession No. GCA_000226075.1) by BLASTn from BLAST 2.6.0+ [7]. After removing baits with hits to the potato genome, a total of 257,046 baits remained. Any baits that overlapped by at least 50% and were 95% identical were clustered together, where one representative of the cluster was retained, reducing the overall number of baits down to 143,847. To generate baits that target non-gene regions, a similar approach was taken, where coordinates for all the regions of the genome outside of gene regions were extracted from each genome and 80 nt baits at the same 0.75× tiling density were simulated, as above. This resulted in 47,542 baits for MB42 and 57,522 baits for LEV6574. Using BLASTn from BLAST 2.6.0+, baits that had exactly 1 hit, corresponding to the region that they were designed from in their respective genomes, were retained which resulted in 29,209 baits MB42 and 29,900 baits for LEV6574. Again, the baits with an overlap of at least 50% and were 95% identical were clustered together where one representative was retained, thereby reducing the number of baits down to 39,231 baits covering non-gene regions. Up to this point, a total of 183,078 baits were designed (143,847 baits in the gene regions plus 39,231 baits in the non-gene regions). To fit the final customized bait set of 180,000 baits (an option offered by Daicel Arbor Biosciences), 3,078 of the baits designed from gene regions with the highest GC content were removed because high GC regions and baits are more likely to form secondary structures, which can reduce capture efficiency (personal communication, Daicel Arbor Biosciences). All bait sequences are available in S1 File.

### Selection of samples

A total of 15 isolates of *S*. *endobioticum* from Europe, Asia and Canada, of pathotypes 1(D1), 2(G1), 6(O1), 8(F1), 18(T1) and 38(Nevşehir) were chosen for testing. The samples from Europe and Asia were handled by the NPPO-NL/WUR group (van de Vossenberg & van der Lee) and the Canadian samples were handled by the AAFC/CFIA group (Nguyen & Antoun). These samples represented spores from compost, purified spores and crude wart tissue derived from tuber galls of infected potatoes (Table 1). Resting spores were isolated from 200 g compost, which serve as inoculum for bio-assays, from the NPPO-NL *S*. *endobioticum* collection with a zonal centrifuge as described previously [5]. For the Canadian material, resting spores were collected in sieves, washed, and centrifuged, as described previously [8–10].

**Table 1:** Metadata of *Synchytrium endobioticum* isolates in this study, their C_q_ values and library pooling strategy.

Lab Sample Code	SendoTrack-ID^a^	Pathotype	Country	Location	Collection Year	Source Material	C_q_^b^	Pool number
MB6	SeTr-074-001	2(G1)	The Netherlands	Groningen, Mussel	1987	Spores from compost	16.3	NPPO-1
pot_ID-127	SeTr-117-001	2(G1)	Germany	Thüringen, Giessübel	1995	Spores from compost	16.3	NPPO-1
MB56	SeTr-071-002	38(Nevsehir)	Turkey	Nevsehir	2005	Spores from compost	16.5	NPPO-1
MB42	SeTr-060-003	1(D1)	The Netherlands	Noord-Brabant, Langenboom	2002	Spores from compost	16.9	NPPO-2
MB70	SeTr-080-001	6(O1)	Germany	?	2007	Spores from compost	16.9	NPPO-2
MB15	SeTr-044-004	18(T1)	Germany	?	1999	Spores from compost	16.9	NPPO-2
pot_ID-151	SeTr-017-001	1(D1)	The Netherlands	Noord-Brabant, Luyksgestel	2011	Spores from compost	17.0	NPPO-3
MB21	SeTr-050-001	6(O1)	The Netherlands	Drenthe, Smilde	2004	Spores from compost	17.1	NPPO-3
pot_ID-189	SeTr-023-003	18(T1)	The Netherlands	Groningen, Alteveer	2013	Spores from compost	17.6	NPPO-3
pot_ID-175	SeTr-129-001	2(G1)	Germany	?	2007	Spores from compost	17.9	NPPO-4
MB69	SeTr-078-002	1(D1)	Sweden	?	2000	Spores from compost	19.2	NPPO-4
MB52	SeTr-067-001	2(Ch1)	Poland	?	2006	Spores from compost	22.4	NPPO-4
CHY1003f	NA	8(F1)	Canada	Prince Edward Island, Baltic	2018	Spores	20.2	AAFC-1
CHY1003g	NA	8(F1)	Canada	Prince Edward Island, Baltic	2018	Crude wart tissue	26.1	AAFC-2
LEV6748f	LEV6748	6(O1)	Canada	Prince Edward Island, New Glasgow	2002	Spores	17.8	AAFC-1
LEV6748g	LEV6748	6(O1)	Canada	Prince Edward Island, New Glasgow	2002	Crude wart tissue	24.2	AAFC-2
LEV6574s	LEV6574	6(O1)	Canada	Prince Edward Island, St. Eleanors	2012	Spores	16.1	AAFC-1
LEV6574t	LEV6574	6(O1)	Canada	Prince Edward Island, St. Eleanors	2012	Crude wart tissue	28.0	AAFC-2
MTL1003a	NA	NA	NA	NA	NA	Control: *Avena sativa*	NA	AAFC-1
WGA NTC	NA	NA	NA	NA	NA	No template control from WGA	NA	AAFC-1

^a^SendoTrack-ID from [5]

^b^C_q_ values of the SSU TaqMan assay from [12], NA = not applicable or not done.

### DNA extraction, whole genome amplification and real-time PCR

DNA was extracted from the European, Asian and Canadian samples following previously published protocols [5]. DNA concentrations were checked using a Qubit Flourometer (Thermo Fisher Scientific, Waltham, Massachusetts, USA). Fragment size distribution was checked using Agilent TapeStation 4200 (Agilent, Santa Clara, California, USA). For the Canadian samples, genomic DNA was subjected to Whole Genome Amplification (WGA) using REPLI-g Single Cell Kit (Qiagen, Germantown, Maryland, USA) according to the manufacturer’s instructions, with 0.9–2.8 μg DNA input per reaction, and purified using ethanol precipitation. For the European and Asian samples, WGA was performed with the GenomiPhi HY Ready-To-Go DNA Amplification Kit (Cytiva, Amersham, Little Chalfont, United Kingdom) according to the manufacturer’s instructions except for using 5 μl input DNA (instead of 2.5 μl). The European and Asian samples were selected with C_t_ values ranging between 18–24 as determined by the ITS TaqMan assay previously described [11]. Subsequently, a 1:400 dilution of the WGA DNA was used in the SSU real time PCR assay [12] to verify the relative quantity of *S*. *endobioticum* DNA in each of the samples (Table 1).

### Library preparation

Whole-genome libraries were prepared from approximately 100 ng WGA DNA using NEBNext Ultra II FS Kit following the protocol for inputs ≤100 ng (New England BioLabs, Ipswich, Massachusetts, USA), with the following modifications: the NEBNext Adaptor for Illumina was replaced with the Universal iTru adapter [13]. The libraries were amplified with iTru i5 and i7 primers [13] at a the final concentration of 0.5 μM, purified using 1x Sera-Mag Select Beads (Cytiva, Amersham, Little Chalfont, United Kingdom), and resuspended in a final volume of 33 μL of 0.1× TE buffer.

### Target enrichment and sequencing

The MyBaits Hybridization Capture for Targeted NGS protocol (Version 5.01, Daicel Arbor Biosciences, Ann Arbor, Michigan) was followed with some modifications. The High Sensitivity option of the protocol was chosen. Prior to enrichment, the libraries were combined in equal amounts (250 ng each) into different pools. Samples with similar expected proportion of target DNA/ similar contamination level were pooled together. Each of the 12 European/Asian samples were also tested as not pooled (i.e. solo enriched). The pools were concentrated using centrifugation and rehydrated in 10 μL of nuclease-free water each. Blocking Mixes recommended for “Most Taxa” were used, with an additional 1 μL of SeqCap EZ Developer Reagent (Roche, Laval, Quebec) to aid in the blocking of plant DNA. The final volume of the Blocking Mix was 6.5 μL, of which 6 μL was mixed with 7 μL of the pooled libraries from above.

Two rounds of hybridization were performed as described in the protocol, with 65°C chosen as the hybridization and wash temperature for the Canadian samples, and 60°C for the European/Asian samples. The reactions were incubated in the thermocycler for 20 h for both rounds. Wash Buffer X preparation, Capture Bead preparation, the binding of beads and hybrids and the washing and resuspension of beads was completed according to the manufacturer’s protocol.

Enriched library bound to beads was used for amplification with 2× KAPA HiFi HotStart Ready Mix (Roche, Laval, Quebec, Canada) and Illumina PCR Primer Cocktail (Illumina, San Diego, California, USA) in a final volume of 50 μL. Fourteen and eight cycles of PCR were used after the first and second round of hybridization, respectively. Amplified libraries were purified using AMPure XP beads (Beckman Coulter Life Sciences, Brea, California USA) and resuspended in a final volume of 23 μL of 0.1× TE. The non-enriched and target enriched libraries were sequenced in two separate Illumina runs. Prior to sequencing, each library was diluted to 4 nM concentration and 5 μL of each was combined into a single pool.

Sequencing of the Canadian samples was performed on an Illumina NextSeq instrument at the Molecular Technologies Laboratory (Ottawa Research and Development Centre, Agriculture and Agri-Food Canada). The final pooled library concentration was 1.6 pM for non-enriched libraries and 1.5 pm for target enriched libraries. Both runs were performed with 1% PhiX control spike-in. Library preparation, target enrichment and sequencing of the European/Asian samples were done at GenomeScan (Leiden, the Netherlands). Reads are available for download on NCBI SRA under BioProject PRJNA1012739.

### Bioinformatics analyses

The quality of the raw reads was assessed with fastqc 0.11.8 (https://www.bioinformatics.babraham.ac.uk/projects/fastqc/). The reformat.sh script from BBTools 38.22 (https://jgi.doe.gov/data-and-tools/bbtools/) was used to count the number of reads and bases sequenced, as well as to subsample libraries to the same number of bases of 1.19 Gb, using the parameter *samplebasestarget = 1190000000*. To measure the number of bases sequenced that belong to *S*. *endobioticum*, the bbmap.sh script from BBTools 38.22 was used to map the subsampled reads to the LEV6574 assembled contigs (NCBI Accession No. QEAM00000000.1), generating a sorted BAM file in the process.

From this point onward, for the European/Asian samples, bioinformatic analyses were performed on the solo enriched data rather than pooled data. T-tests were performed using the Analysis ToolPak in Microsoft Excel. Other pertinent statistics were obtained from the sorted BAM file to compare enriched vs non-enriched samples. First, bedtools v2.30.0 [14] was used to generate coordinates of 1000 bp window of each contig of LEV6574. The bedcov program in samtools v1.9 [15] was used to calculate: 1) base count from mapped reads; 2) number of bases in the window having a depth above 0; 3) number of reads mapped in each of the 1000 bp windows. The average coverage of each window was calculated by taking the (1) base count from mapped reads in that window and dividing it by 1000 bp (S2 Table). The proportion of the window covered by a read was calculated by taking (2) the number of bases having a depth above 0 and dividing it by 1000 bp (S3 Table). Violin plots were generated with R 4.1 (https://www.R-project.org/).

Genome assembly was performed with MEGAHIT v1.1.4 [16] with default parameters (k = 21, 29, 39, 59, 79, 99, 119, 141). Assembled contigs were searched against a local whole-genome database of plants, fungi that include *S*. *endobioticum* LEV6574, oomycetes, and bacteria (downloaded from trusted sources such as NCBI RefSeq, Joint Genome Institute [JGI] MycoCosm, and Ensembl) with BLASTn from BLAST 2.12.0+ [7], with an e-value threshold of e^−100^. Contigs showing a hit to *S*. *endobioticum* were considered putative *S*. *endobioticum* and were isolated into a separate file. QUAST 5.0.2 was used to summarize genome assembly statistics [17]. Assembly lengths are summarized in S4 Table.

To detect if and how many *S*. *endobioticum* specific genes could be found in the genome assemblies, a script (see S2 File) was used to extract all 8671 gene sequences (introns/exons were included) from the *S*. *endobioticum* LEV6574 GenBank record QEAM00000000.1 as a fasta file. The extracted gene sequences were used as the query for the program BLASTn from BLAST 2.12.0+ to search for similar sequences in all genome assemblies, with a e-value threshold of e^−100^. S5 Table shows the percentage of the 8671 *S*. *endobioticum* LEV6574 genes putatively detected in the genome assemblies.

Scripts used for all bioinformatic analyses are available in S2 File.

## Results

### Samples

A total of 12 European/Asian samples, representing DNA extracted from spores from compost, were chosen. These represented pathotypes 1(D1), 2(G1), 2(Ch1), 6(O1), 18(T1), and 38(Nevşehir). For the Canadian samples, we chose 3 different isolates, representing pathotypes 8(F1) and 6(O1), where the DNA was extracted either from purified spores or crude wart tissue, for a total of 6 samples. We also included 2 controls: DNA from *Avena sativa* as input; a no DNA template control from the whole genome amplification step. The real time PCR assay showed C_q_ values ranging from 16.1 (containing the most *S*. *endobioticum* DNA) to 28.0 (containing the least *S*. *endobioticum* DNA). For the European samples, target enrichment was performed on individual samples (solo enriched) but also by combining samples with similar C_q_ values in the same pool (pooled enriched), resulting in four different pools (Table 1, NPPO-1, NPPO-2, NPPO-3, and NPPO-4). For the Canadian samples, the spore and crude wart tissue samples were pooled separately (Table 1, AAFC-1 and AAFC-2).

### Sequencing output and re-sampling of data

Each sample gave varying number of reads and bases sequenced (S1 Table). To assess the number of bases sequenced that belong to *S*. *endobioticum*, we subsampled each dataset to roughly the same number of bases sequenced. We chose a minimum of 1.19 Gb of bases sequenced as the cut-off and excluded two European pooled target enriched samples (MB52 & pot_ID-127) for subsequent analysis.

**Fig 1:**
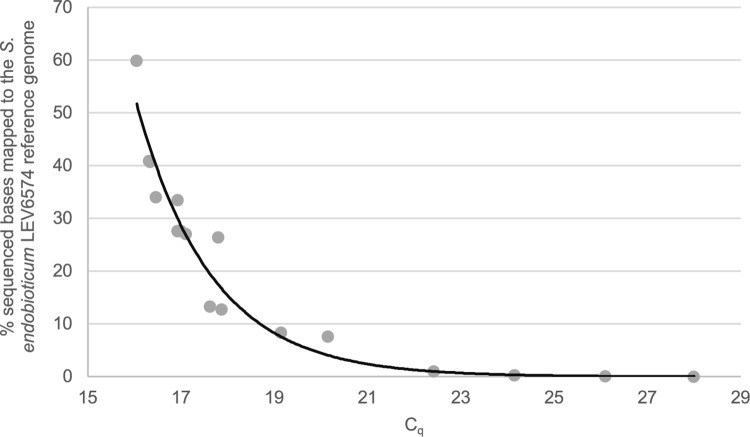
Correlation of the percentages of bases mapped to the *S*. *endobioticum* genome and C_q_ value of non-enriched samples tested. The exponential relationship is defined as y = 1E+06e^-0.621x^ with R^2^ = 0.94.

Once the datasets were subsampled to 1.19 Gb of bases sequenced, we mapped each of them to the *S*. *endobioticum* LEV6574 reference genome from [4] (S1 Table). In the non-enriched samples, we noticed that those with lower C_q_ values gave more bases mapped to LEV6574 compared to those with higher C_q_ values, as expected, because samples with lower C_q_ values have more *S*. *endobioticum* DNA compared to those with higher C_q_ values. There is a correlation (R^2^ = 0.94), where increasing C_q_ values will result in an exponential decline percent of bases sequenced that mapped to the *S*. *endobioticum* reference genome (Fig 1).

In samples with lower DNA of *S*. *endobioticum* (higher C_q_ values), target enrichment was effective at increasing the number of bases mapped to *S*. *endobioticum* (S1 Table). The same effect was observed in the Canadian samples from crude wart tissue, which we consider to be a difficult class of samples as these contain DNA from a range of other microorganisms and the potato host. We observed the non-enriched crude wart tissue samples yielded less than 1% of bases mapped, but when the same sample was subjected to target enrichment, the number of reads mapped to *S*. *endobioticum* were increased between 290.7-fold to 783.3-fold.

When comparing the percent of bases mapped of the non-enriched samples to the pooled enriched samples using a paired t-test, the difference was statistically significant (P < 0.001). In contrast, the pooling of samples did not affect the enrichment efficacy. When comparing the European/Asian pooled enriched and solo enriched samples, the percent of bases mapped to LEV6574 were similar, ranging from roughly 81% to 89%. When we compared pooled enriched and solo enriched samples using a paired t-test, the difference was not statistically significant (P = 0.7). All calculations for t-tests are shown in S3 File. These results show that the performance of target enrichment is not affected by pooling.

### Coverage

**Fig 2:**
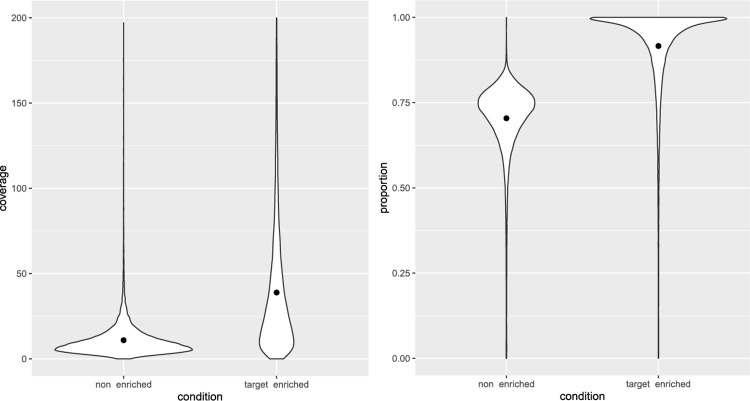
Violin plots showing the average coverage in each 1000 bp window, as well as the proportion of this window that is covered by at least one read, in non-enriched and target enriched samples. The black dot inside each histogram indicates the mean value.

We mapped all datasets to the *S*. *endobioticum* LEV6574 genome and looked at coverage statistics in each 1000 bp window to further assess effectiveness of target enrichment. We calculated the average coverage in each 1000 bp window, as well as the proportion of this window that is covered by at least one read (Fig 2, S2 and S3 Tables). When it comes to average coverage, the non-enriched samples had a lower value (average = 12, median = 8) compared to enriched samples (average = 44, median = 38). When it comes to the proportion of the window covered by at least one read, the non-enriched samples also had a lower value (average = 0.70, median = 0.91) compared to the target enriched samples (average = 0.92, median = 0.94). There will always be areas of the genome that will be missed or have low coverage, or unusually high coverage (e.g. unresolved repeats) during sequencing. However, target enrichment can enhance overall coverage.

### Genome assembly & *S*. *endobioticum* genes detected

**Fig 3:**
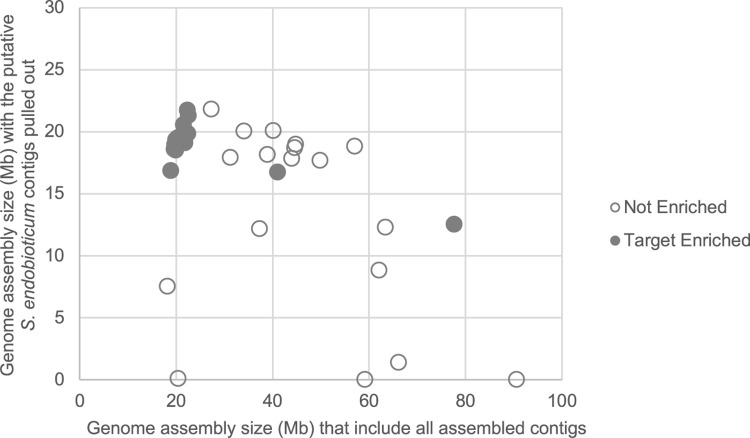
Scatter plot comparing the genome assembly size of all assembled contigs against the genome size produced from pulling out putative *S*. *endobioticum* contigs, in non-enriched and target enriched samples.

We used a metagenome assembler to assemble the 1.19 Gb subsampled non-enriched and target enriched datasets (S4 Table). On average, the non-enriched samples yielded total assemblies of 46 Mb on average while the target enriched samples yielded a smaller assembly size of only 25 Mb. After identifying putative *S*. *endobioticum* contigs and pulling them out, we obtained total assembly sizes of only 12.9 Mb for non-enriched samples, while the target enriched samples was larger at 18.9 Mb on average. When comparing the genome size of all assembled contigs against the genome size produced from pulling out putative *S*. *endobioticum* contigs, the target enriched samples formed a tight cluster around 20 Mb (the expected genome size of *S*. *endobioticum*) while the non-enriched samples were much more dispersed, with some samples showing no putative *S*. *endobioticum* sequences in the assembly (Fig 3). This indicates target enrichment biased genome assembly towards *S*. *endobioticum* sequences, yielding smaller assembly sizes but higher representation of putative *S*. *endobioticum* contigs with less sequences from off-target organisms.

We detected *S*. *endobioticum* specific genes in those assemblies by BLASTn. Target enrichment boosted the number of *S*. *endobioticum* genes detected (S5 Table). For example, in the Canadian samples from the crude wart tissue (CHY1003f, LEV6574t, LEV6748g), the genes detected were 0%-4% when not enriched, but this number increased to 87%-98% when subjected to target enrichment. The European sample MB52, with a very low amounts of *S*. *endobioticum* DNA (Table 1), saw a similar effect where 22% genes were detected when not enriched and 90% genes were detected when target enriched.

## Discussion

In this study, we designed a protocol that improves the recovery of *S*. *endobioticum* nuclear genome sequences from Illumina library preparations, especially in cases where the starting amount of *S*. *endobioticum* DNA is low or the samples are highly contaminated. The bait design strategy involved removing baits with homology to the potato genome to reduce non-target binding and improve specificity. By using both gene and non-gene regions as targets, a comprehensive set of 180,000 baits was created, allowing for efficient enrichment of *S*. *endobioticum* nuclear genome sequences.

The results showed the efficacy of the target enrichment protocol in enhancing the recovery of *S*. *endobioticum* sequences from diverse sample types and from various pathotypes. Notably, target enrichment significantly increased the number of bases sequenced belonging to *S*. *endobioticum*. This effect was particularly prominent in challenging samples, such as those derived from crude wart tissue or those with low starting amounts of *S*. *endobioticum* DNA. We observed that the pooling of samples did not affect target enrichment efficacy. Therefore, we recommend pooling whenever possible to reduce molecular bait usage, lowering the cost of experiments. The average coverage and proportion of the genome covered by at least one read were consistently higher in target enriched samples compared to non-enriched ones. Genome assembly results indicated that the target enrichment protocol biased the assembly towards *S*. *endobioticum* sequences, resulting in smaller assembly sizes but higher representation of putative *S*. *endobioticum* contigs. Finally, target enrichment increased the number of *S*. *endobioticum* genes detected in those genome assemblies. Improvements in coverage, genome assembly and *S*. *endobioticum* genes recovery could be useful when studying regions with biological relevance, such as effector genes linked to pathotype identity, single nucleotide polymorphisms or indels. The baits were designed using the genomes of *S*. *endobioticum* MB42 (pathotype 1(D1)) and LEV6574 (pathotype 6(O1)) and unique genes and effectors potentially found in other pathotypes could be missed by our current approach. As a future study, we intend to develop a new bait set that would be built based on genome sequences of the most commonly found pathotypes, which should be better at capturing more *S*. *endobioticum* specific effectors.

To our knowledge, a target enrichment protocol to enhance next generation sequencing, at the whole genome level, of obligate fungal and oomycete plant pathogens has not been attempted before. Many of the applications of target enrichment are designed to recover some genomic loci (e.g. partial genomes) and normally applied to broad range of species, rather than one species [3]. So for the first time, we successfully designed and implemented a hybridization-based target enrichment protocol to enhance the recovery of an obligate plant pathogen’s nuclear genome sequences. It is important to note that all DNA samples we tested were whole genome amplified prior to target enrichment. The bait set can capture nuclear sequences of a broad range of *S*. *endobioticum* pathotypes. This tool can be used to easily sequence samples with low amounts of *S*. *endobioticum* DNA (e.g. water samples, herbarium samples), as well as enhance NGS based molecular detection methods (e.g. metagenomics, targeted gene panel detection).

## Supporting information

S1 Table Sequencing outputs and mapping statistics of samples. (XLSX)

S2 Table Coverage of each 1kb window. (XLSX)

S3 Table Proportion of 1kb window covered by reads. (XLSX)

S4 Table Metagenome assembly lengths. (XLSX)

S5 Table Percentage of the 8671 *S*. *endobioticum* LEV6574 genes putatively detected on the genome assemblies by BLASTn. (XLSX)

S1 File Bait sequences. (ZIP)

S2 File Scripts used for bioinformatic analyses. (ZIP)

S3 File Calculations for t-tests. (XLSX)
