# Correction: A target enrichment approach for enhanced recovery of *Synchytrium endobioticum* nuclear genome sequences

**DOI:** 10.1371/journal.pone.0310148

**Published:** 2024-09-04

**Authors:** Hai D. T. Nguyen, Ekaterina Ponomareva, Kasia Dadej, Donna Smith, Melissa Antoun, Theo A. J. van der Lee, Bart T. L. H. van de Vossenberg

## Notice of Republication

An incorrect copyright license was mistakenly applied to this article [1] upon publication. The article was republished on August 27, 2024, to update the Copyright license and statement. Please download this article again to view the correct version.
